# Gastroesophageal varices in primary biliary cholangitis with anti-centromere antibody positivity: Early onset?

**DOI:** 10.1515/biol-2022-0979

**Published:** 2024-11-19

**Authors:** Han Shi, Qi Wang, Hui Liu, Bin Xu, Yanmin Liu, Juan Zhao, Lina Sun, Dexi Chen, Chunyang Huang, Ronghua Jin

**Affiliations:** Beijing Institute of Hepatology, Beijing You’an Hospital, Capital Medical University, 8 Xitoutiao, Youanmenwai Street, Fengtai District, Beijing, China; Beijing Key Laboratory of Emerging Infectious Diseases, Institute of Infectious Diseases, Bejing Ditan Hospital, Capital Medical University, 8 Jingshundong Street, Chaoyang District, Beijing, PR. China; Beijing Institute of Infectious Diseases, Beijing, PR. China; Clinical Pathology Center, Beijing You’an Hospital, Capital Medical University, 8 Xitoutiao, Youanmenwai Street, Fengtai District, Beijing, China; National Center for Infectious Diseases, Beijing Ditan Hospital, Capital Medical University, 8 Jingshundong Street, Chaoyang District, Beijing, PR. China; Second Department of Liver Disease Center, Beijing You ‘an Hospital, Capital Medical University, 8 Xitoutiao, Youanmenwai Street, Fengtai District, Beijing, China; Changping Laboratory, Beijing, PR. China

**Keywords:** primary biliary cholangitis, anti-centromere antibodies, gastroesophageal varices, portal hypertension, laboratory tests, liver biopsies

## Abstract

Primary biliary cholangitis (PBC) is an autoimmune liver disease. During the diagnostic process, the patient’s autoimmune antibodies are routinely examined. Approximately 20% of PBC patients have positive anti-centromere antibody (ACA). We evaluated the clinical characteristics of ACA-positive and ACA-negative PBC patients to explain the differences in disease progression between these two groups. Retrospective data from 961 PBC patients at Beijing Youan Hospital from 2010 to 2019 were gathered and separated into two groups based on ACA positivity. We collected and evaluated clinical laboratory indices, gastroscopy findings, and liver function assessments. In addition, 60 liver biopsies were available for comparison between the 2 groups. Pathologists staged the histological findings using the Ludwig staging criteria and Nakanuma staging and grading. Immunohistochemical staining was also performed on liver biopsies to examine the expression of cytokeratin 7 (CK7) in the tissue. A synthesis of clinical indicators in the large cohort showed that alanine transaminase, aspartate aminotransferase, total bilirubin, IgG, white blood cell, and platelet were significantly lower in the ACA-positive group, indicating that the overall status of liver injury was more moderate in the ACA-positive group. Additionally, ACA-positive patients in the non-cirrhotic group were more likely to present with gastroesophageal varices related to portal hypertension. Finally, analysis of pathologic findings showed that parameters were mostly comparable in the two groups, but CK7 differed and was more significantly lower in the ACA-positive group in albumin–bilirubin grade 2 and 3 patients. In summary, we characterized and compared the clinical features of ACA-positive and ACA-negative PBC patients, corroborating previous studies on the relationship between ACA positivity and portal hypertension cross-sectionally. It suggested that gastroesophageal varices might happen in the earlier course of PBC natural progression in the ACA-positive group.

## 1 Introduction

Primary biliary cholangitis (PBC) is an autoimmune liver disease that is detected more often in women over the age of 40 years. Patients are usually asymptomatic in the early stage of PBC, while some may gradually begin to show unspecific symptoms, including fatigue and pruritus. Eventually, the disease may progress into liver fibrosis and cirrhosis. For diagnosis of PBC, laboratory tests of liver function can present elevated alkaline phosphatase (ALP) and/or gamma-glutamyltranspeptidase (GGT). To date, the etiology and pathogenesis of PBC are not yet fully understood. An autoimmune antibody test is also an effective diagnostic approach [1–3]. Approximately 90% of PBC patients exhibit anti-mitochondrial antibody (AMA) serological positivity, a certain proportion of whom also present with anti-nuclear antibody (ANA) positivity [1]. Previous reports proved that AMA positivity did not seem to be correlated with the severity and prognosis of PBC, while distinct immunofluorescent nuclear patterns of ANA, for instance, anti-Gp210 and anti-Sp100 antibody, have prognostic value for PBC patients [4,5]. In the ANA test, the anti-Sp100 antibody manifests multiple nuclear dots pattern, and the anti-Gp210 antibody gives a punctate nuclear envelope pattern. Studies have proven that the anti-gp210 antibody is related to poorer ursodeoxycholic acid (UDCA) treatment response and prognosis [6]. Anti-centromere antibody (ACA), manifesting centromeric pattern in ANA test, exhibits positive in 20–30% of PBC patients, which has been demonstrated to have an increased risk of developing portal hypertension [7].

Liver biopsy continues to play an important role in the diagnosis of PBC in certain special circumstances of AMA or cholestatic proof absence, and in ruling out other coexisting liver diseases [1,3]. The classical Ludwig staging system is used to classify PBC liver biopsies into four stages based on manifestations [8–10]. Moreover, a new staging and grading system has recently been suggested by Nakanuma and has been validated in other research, which mainly focuses on fibrosis, bile duct loss, chronic cholestasis, and necro-inflammatory activity [11,12].

Since the number of ACA-positive PBC patients is only a small fraction of all PBC cases, there are limited cross-sectional studies about the clinical features of ACA-positive PBC patients based on the large sample population. Besides, the differences between the ACA-positive and negative groups have not been fully interpreted on the basis of pathologic findings. To illuminate this uncharted area, we compared the clinical information and pathological features of PBC patients from these two groups and evaluated their gastroscopy results, especially for non-cirrhotic PBC patients through a large collection of samples. Laboratory tests of liver function revealed that indicators like ALP and GGT predicting cholestasis were comparable in both groups, while indicators on liver cell injury like aspartate aminotransferase (AST) and alanine transaminase (ALT) and prognosis were milder in the ACA-positive group. We also analyzed the differences between the two groups after categorizing whether the condition is cirrhotic or not, finding that ACA positivity positively correlated with gastroesophageal varices. Finally, the liver biopsy results were also consistent with our findings described above upon analysis, which revealed that cytokeratin 7 (CK7) was lower in the ACA-positive group and had milder ductular destruction.

## 2 Methods

### 2.1 Study design and participants

Patient information was retrospectively collected from the hospital outpatient management system of Beijing Youan Hospital affiliated with Capital Medical University from 2010 to 2019, including clinical laboratory tests and imaging and gastroscopy findings. Laboratory tests covered blood cell counts, liver function parameters, autoimmune antibodies, and immunoglobulin. The baseline albumin–bilirubin (ALBI) grade was calculated to evaluate the prognosis [13]. This grade has been shown in several studies to have the ability to assess prognosis in patients with PBC as well, with the lower its score, the better the prognosis [14,15]. The diagnostic criteria for PBC, based on the European Association for the Study of the Liver clinical practical guidelines published in 2017, were as follows: (a) signs of chronic cholestasis (elevated ALP and GGT); (b) AMA and/or AMA-M2 subtype positivity; and (c) liver biopsy showing pathological changes related to PBC; patients meeting at least 2 of the above criteria were included. The exclusion criteria were (a) other current viral hepatitis, drug-induced liver injury, alcoholic liver disease, or nonalcoholic fatty liver disease; (b) hepatocellular carcinoma or liver metastatic carcinoma; and (c) severe heart or kidney failure or other organic diseases such as severe pulmonary disease or advanced cancer. Patients with cirrhosis were further scored 1–3 in order of severity by the Child-Pugh scale after assessing bilirubin and albumin (ALB) levels, prothrombin time (PT) (or the international normalized ratio), and encephalopathy and ascites status. These scores were then summed as follows: Child-Pugh A: 5–6; Child-Pugh B: 7–9; and Child-Pugh C: 10–15 [16]. The study was conducted according to the 1975 Declaration of Helsinki. All the participating institutional research committees approved the protocol under local regulations.


**Informed consent:** Informed consent has been obtained from all individuals included in this study.
**Ethical approval:** The research related to human use has been complied with all the relevant national regulations, institutional policies, and in accordance with the tenets of the Helsinki Declaration and has been approved by the Ethics Committee of Capital Medical University affiliated with Beijing You’an Hospital (No. LL-2019-128-K).

### 2.2 Autoimmune antibody detection

Mainly AMA and ANA were examined in all PBC patients. Both AMA and ANA were detected using indirect immunofluorescence (IIF) on HEp-2 cells. ANA showed distinct patterns on HEp-2 cells by IIF, including ACA, anti-sp100, and anti-gp210 antibodies. ACA produced a centromeric pattern characterized by discrete coarse speckles scattered in interphase cells and aligned at the chromatin on mitotic cells. Anti-Sp100 antibody gave an ANA pattern called multiple nuclear dots showing countable discrete nuclear speckles (6–20 nuclear dots/cell). Anti-Gp210 antibody gave an ANA pattern showing punctate staining on the nuclear envelope in interphase cells, with accentuation of fluorescence at the points where adjacent cells touch each other and no staining of the metaphase and anaphase chromatin plates [17].

### 2.3 Histological scoring and staining

Sixty liver biopsies were retrospectively collected from the patients in 2018–2019. All liver biopsy samples were stained using hematoxylin and eosin and processed by immunohistochemical staining to assess CK7 expression. Histological evaluation of the liver biopsy samples was performed according to Ludwig and Scheuer’s staging system [3,7] and the Nakanuma staging and grading system. The Nakanuma staging system includes fibrosis and bile duct loss on histological sections, which are scored 0–3 in the order of severity of lobules or portal tracts involved. The Nakanuma grading system focuses on necro-inflammatory activity, which includes cholangitis activity (CA) and hepatitis activity (HA) and is also scored 0–3 in order of severity. The CA score is accessed by the number of portal tracts involved in the histological sections, while HA is accessed by the extent of interface hepatitis [10]. We compared all Nakanuma scale scores by weighting them with the results of the Ludwig stage.

### 2.4 Statistical analysis

Normally distributed continuous variables are presented as the mean ± standard deviation and were compared with the independent *t*-test for two samples. Continuous variables that were not normally distributed are described with the median (interquartile range) and were compared between groups with the Mann‒Whitney *U* test. For categorical variables, the number and proportion (%) are shown, and the Pearson chi-squared test was used for comparison between groups. Logistic regression was used for multivariable analysis. Statistical analyses were performed by the IBM SPSS Statistics program (Version 25.0) and Prism (Version 9.4.1). A *p* value of <0.05 was considered statistically significant.

## 3 Results

### 3.1 Overall clinical characteristics of the study patients

There were totally 961 PBC patients meeting the inclusion and exclusion criteria above, of whom 254 patients were ACA positive and 707 patients were ACA negative. Statistical analysis showed that women were more associated with ACA positivity (93.7% vs 86.3%, *p* < 0.05). Descriptive clinical information and comparisons between the two groups are expressed in Table 1. Comparing the clinical information of ACA negative and positive groups, the outcome indicated that the difference in age was not statistically meaningful. The indicators reflecting the degree of inflammation and necrosis of liver parenchyma (AST, ALT) were significantly lower in the positive group than in the negative group, while the indicators representing bile duct damage (ALP, GGT) and hepatic synthetic function (ALB, PT) were at comparable levels in the two groups. As one of the important prognostic indicators, bilirubin in the ACA-positive group was significantly lower than that in the negative group, without any remarkable difference demonstrated by the ALBI grade [18].

**Table 1:** Clinical characteristics of ACA-positive and ACA-negative groups

	ACA negative (*N* = 707)	ACA positive (*N* = 254)	*p* value
Total (*N* = 961)			0.0024^1^
Gender (*n* (%))			
Female	610(86.3%)	238(93.7%)	
Male	97(13.7%)	16(6.3%)	
Age (years)	56.2(10.9)	57.3(10.8)	0.169^2^
ALT (U/L)	48.4(62.45)	37.3(48.68)	0.002^3^
AST (U/L)	61.3(68.6)	48.2(41.13)	<0.001
AST/ALT	1.15(0.85)	1.19(0.72)	0.452
TBIL (μmol/L)	22.1(28.25)	18.5(13.88)	<0.001
GGT (U/L)	170.3(269.7)	161.45(256.03)	0.071
ALP (U/L)	190.3(180.05)	194.35(163.43)	0.831
ALB (g/L)	40.5(10.1)	39.8(9.4)	0.259
IgA (g/L)	3.29(2.06)	3.27(1.95)	0.707
IgG (g/L)	17.1(6.8)	15.9(5.28)	0.000
IgM (g/L)	3.21(3.2)	2.93(2.31)	0.175
WBC (×10^9^/L)	5.05(2.6)	4.43(2.49)	<0.001
PLT (×10^9^/L)	157(135.5)	137(125)	0.001
PT (s)	11.3(2.15)	11.3(2.07)	0.211
ALBI grade			0.197
1	322(45.7%)	121(53.8%)	
2	300(42.6%)	77(34.2%)	
3	82(11.6%)	27(12%)	
Varices (*n* = 317)			0.097^4^
None	57(25.8%)	17(17.7%)	
Mild	44(19.9%)	18(18.8%)	
Moderate	19(8.6%)	9(9.3%)	
Severe	101(45.7%)	52(54.2%)	
PHG (*n* = 235)			0.058
No	47(78.3%)	114(65.1%)	
Yes	13(21.7%)	61(34.9%)	
Cirrhotic patients (*n* = 102)			0.012
Child-Pugh A	13(9.8%)	2(4.3%)	
Child-Pugh B	81(61.4%)	43(93.5%)	
Child-Pugh C	38(28.8%)	1(2.2%)	

Data were expressed as *n* (%) and median (interquartile). Abbreviations: alanine aminotransferase; AST: aspartate aminotransferase; TBIL: total bilirubin; GGT: gamma-glutamyltranspeptidase; ALP: alkaline phosphatase; ALB: albumin; WBC: white blood cell; PLT: platelet; PT: prothrombin time. S: seconds. ALBI: albumin–bilirubin ^1^Pearson chi-squared test. ^2^Independent *t*-test for two samples. ^3^Mann‒Whitney *U* test. Reference range: ALT 7–40 U/L; AST 13–35 U/L; TBIL 5–21 μmol/L; ALB 40–55 g/L; GGT 7–45 U/L; ALP 50–79 U/L; IgG 7.0–16.0 g/L; IgA 0.7–4.0 g/L; IgM 0.4–2.3 g/L; WBC 3.5–9.5 × 10^9^/L; PLT 120–350 × 10^9^/L; PT 9.9–12.8 s.

A summary of 317 gastroscopy results was collected and analyzed for variceal status and portal hypertensive gastropathy (PHG). The variceal status was classified into no varices, mild varices, moderate varices, and severe varices. Overall, there was no difference at the statistical level between the two groups in terms of gastroesophageal varices. In addition, based on our comprehensive evaluation of imaging and liver function results, there were 178 PBC patients who had progressed into liver cirrhosis status. We classified these liver cirrhosis patients according to Child-Pugh criteria. In patients who already progressed into cirrhosis, the proportion of the ACA-positive group was higher than the negative group on Child-Pugh B (93.5% vs 61.4%, *p* < 0.05).

### 3.2 Comparison of ACA-negative and -positive groups after categorizing cirrhotic status

We then compared the characteristics between the two groups based on whether patients progressed into cirrhosis (Table 2). For non-cirrhotic patients, we found that indicators were statistically less significant between the two groups except AST, total bilirubin (TBIL), and IgG, which were markedly lower in the ACA-positive group (*p* < 0.05). ALT levels were close to being statistically different and also showed a tendency to be lower in the ACA-positive group (*p* = 0.51). For patients progressing into cirrhosis, parameters including ALT, AST, IgG, white blood cell (WBC), and PT all showed notably lower levels (*p* < 0.05). Moreover, the results revealed that ALBI grades were statistically different between the two groups. The median level of ALBI grade was lower in the ACA-positive group than in the negative group (*p* < 0.05). In fact, most of the statistical comparisons between the two groups were more similar and consistent with the findings in overall patient analysis. Differences in WBC and PT were seen in the ACA-positive versus -negative group in cirrhotic patients compared with non-cirrhotic. A lower WBC may reflect a state of portal hypertension hypersplenism, and a lower PT rather suggests a better severity of liver disease state compared with the negative group.

**Table 2:** Comparison between two groups after categorizing cirrhotic status

	Non-cirrhotic patients			Cirrhotic patients		
	ACA negative	ACA positive	*p* value	ACA negative	ACA positive	*p* value
Gender			0.013^1^			0.095
Male	56(13.7%)	7(5.5%)		30(12.8%)	7(6.7%)	
Female	354(86.3%)	120(94.5%)		205(87.2%)	98(93.3%)	
Age	54.4(10.2)	54.6(10.2)	0.796^2^	59.2(10.66)	60.9(10.4)	0.815
ALT	69.4(67.65)	46.6(53.4)	0.051^3^	42.4(55.10)	29.6(24.85)	0.002
AST	72.6(81.15)	54.0(54.4)	0.012	67.1(68.4)	41.8(31.0)	<0.001
TBIL	28.9(34.95)	15.6(13.6)	0.003	32.8(43.0)	20.25(14.65)	<0.001
GGT	332.7(432.1)	210.8(319.7)	0.087	137.1(213.5)	99.9(166.93)	0.077
ALP	241.2(284.0)	208.6(302.7)	0.499	190.8(173.1)	171.95(169.48)	0.463
ALB	38.8(6.85)	40.6(7.2)	0.133	34.5(9.8)	35.25(8.95)	0.099
IgA	2.97(2.44)	3.15(1.72)	0.548	3.82(2.78)	3.495(2.62)	0.921
IgG	17.3(7.05)	14.8(4.6)	0.012	18.7(7.5)	16.55(5.02)	<0.001
IgM	4.34(4.53)	3.5(3.9)	0.397	3.26(3.16)	2.725(2.11)	0.065
WBC	4.95(2.4)	4.0(2.13)	0.113	4.02(2.77)	3.365(1.83)	0.002
PLT	157.0(113.0)	145.0(98.0)	0.291	99.0(71.0)	90.0(56.0)	0.283
PT	11.0(1.65)	11.3(1.9)	0.500	12.7(3.4)	12.0(2.15)	0.001
ALBI grade			0.88			0.004
1	281(68.5%)	87(68.5%)		37(15.7%)	22(21.0%)	
2	113(27.6%)	38(29.9%)		134(57.0%)	71(67.6%)	
3	16(3.9%)	2(1.6%)		64(27.3%)	12(11.4%)	

Data were expressed as *n* (%) and median (interquartile). Abbreviations: alanine aminotransferase; AST: aspartate aminotransferase; TBIL: total bilirubin; GGT: gamma-glutamyltranspeptidase; ALP: alkaline phosphatase; ALB: albumin; WBC: white blood cell; PLT: platelet; PT: prothrombin time; ALBI: albumin–bilirubin. ^1^Pearson chi-squared test. ^2^Independent *t*-test for two samples. ^3^Mann‒Whitney *U* test. Reference range: ALT 7–40 U/L; AST 13–35 U/L; TBIL 5–21 μmol/L; ALB 40–55 g/L; GGT 7–45 U/L; ALP 50–79 U/L; IgG 7.0–16.0 g/L; IgA 0.7–4.0 g/L; IgM 0.4–2.3 g/L; WBC 3.5–9.5 × 10^9^/L; PLT 120–350 × 10^9^/L; PT 9.9–12.8 s.

### 3.3 ACA-positivity was positively correlated with gastroesophageal varices

To verify previous findings that ACA can be a predictor of portal hypertension in non-cirrhotic patients, we drew on uni- and multivariate analyses using logistic regression (Table 3). ACA positivity was the risk factor of gastroesophageal varices (odds ratio [OR] = 3.482, 95% confidence interval [CI] 1.19–10.196, *p* < 0.05), while platelet (PLT) was the protective factor (OR = 0.983, 95% CI 0.976–0.991, *p* < 0.05).

**Table 3:** Uni- and multi-variate analysis of gastroesophageal varices in non-cirrhotic patients

	Univariable analysis	Multivariable analysis					
	*p* value	*B*	SE	Wald	OR (95% CI)		*p* value
Gender	0.281						
Male							
Female							
ACA	<0.05						
Negative							
Positive		1.248	0.548	5.188	3.482 (1.19–10.186)		0.023^1^
Age	<0.05	0.018	0.027	0.453	1.1019 (0.965–1.075)		0.501
ALT	<0.05	−0.01	0.007	2.341	0.99 (0.976–1.003)		0.126
AST	0.053	0.007	0.007	1.051	1.007 (0.994–1.02)		0.305
TBIL	0.501						
GGT	0.254						
ALP	0.879						
ALB	0.19						
IgA	0.215						
IgG	0.304						
IgM	0.491						
WBC	0.086						
PLT	<0.05				0.983 (0.976–0.991)		<0.001
PT	0.595						
AMA	0.108						
Negative							
Positive							
GP210	0.726						
Negative							
Positive							
SP100	0.558						
Negative							
Positive							

Data were expressed as *n* (%) and median (interquartile). Abbreviations: ACA: anti-centromere antibody; alanine aminotransferase; AST: aspartate aminotransferase; TBIL: total bilirubin; GGT: gamma-glutamyltranspeptidase; ALP: alkaline phosphatase; ALB: albumin; WBC: white blood cell; PLT: platelet; PT: prothrombin time; AMA: anti-mitochondrial antibody; ALBI: albumin–bilirubin. ^1^binomial logistic regression analysis was used. The dependent variable was the occurrence of varices.

### 3.4 Comparison of histological manifestations

**Figure 1:**
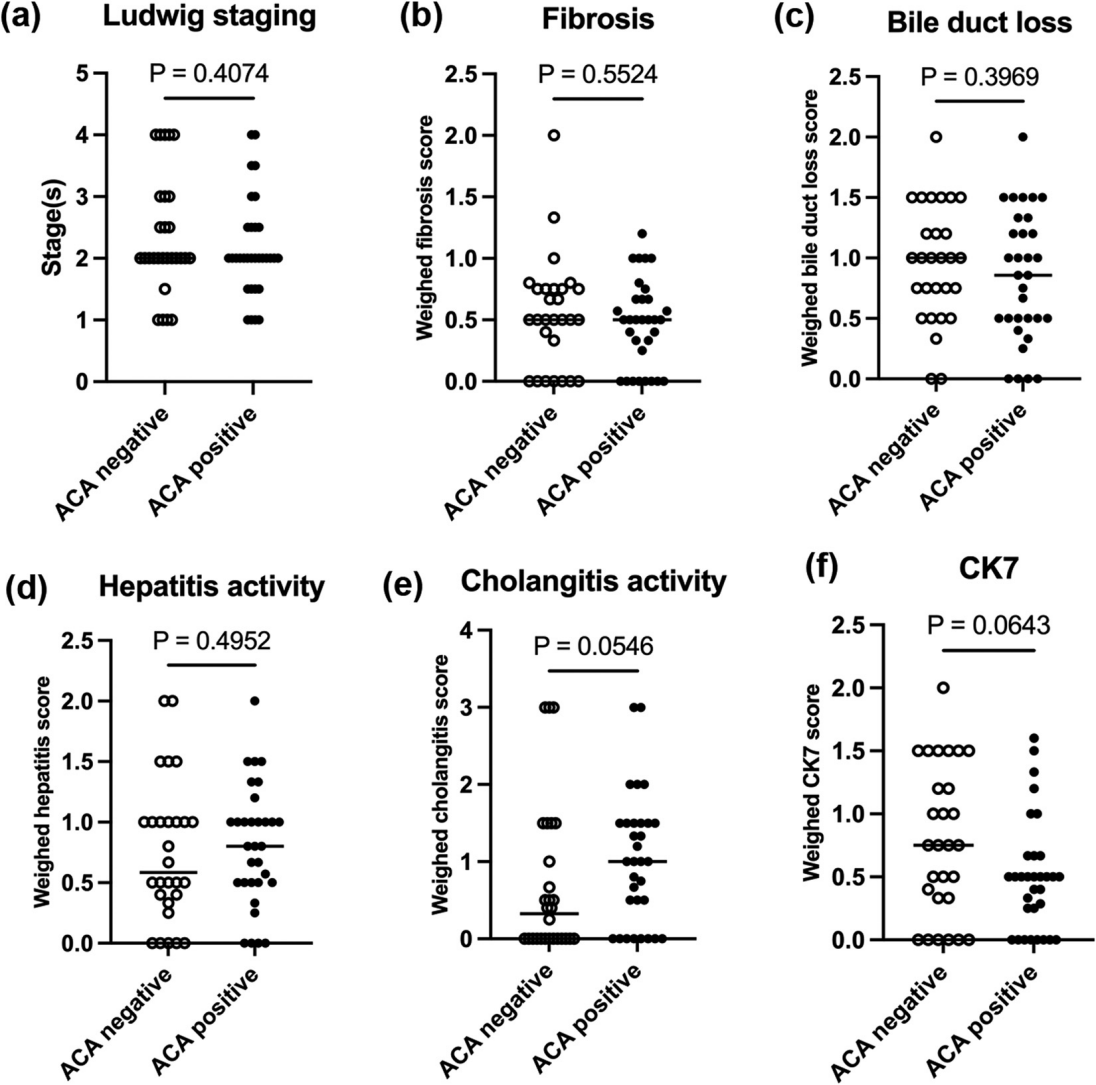
Comparison of Ludwig stages and Nakanuma staging scales between the ACA-positive and -negative group. (a) Comparison of Ludwig stages. (b–e) Comparison of Nakanuma staging and grading scales (fibrosis, bile duct loss, HA, and CA) weighted by Ludwig stages. (f) Comparison of CK7 score weighted by Ludwig stages. Mann–Whitney *U* test was used.

To further investigate the specific differences in the progression of the disease between the two groups, liver biopsies from 60 PBC patients were collected, of which 28 were in the ACA-negative group and 32 were in the ACA-positive group. After all biopsies were rated and scored with the Ludwig system and the Nakanuma system by pathologists, we first compared the scores of the systems between the two groups, including all Nakanuma system rating scales, which were weighted by the Ludwig stages. CK7 is an intermediate filament expressed in the biliary epithelial cells in normal livers; in PBC patients, it is also expressed in hepatocytes. Since the expression of CK7 could reflect the condition known as “ductular metaplasia” in hepatocytes as well as cholestasis status in the progression of PBC patients, we specifically focused on CK7 expression between the two groups [19,20]. Mann‒Whitney *U* tests showed that the differences in the Ludwig stages between the groups were statistically less significant (Ludwig, *p* = 0.407; Nakanuma, *p* = 0.059). Regarding Nakanuma staging and necro-inflammatory grading, the detailed rating scale (fibrosis, bile duct loss, HA, and CA) score was also not significantly different between the two groups (Figure 1b–e), with identical results for immunohistochemical staining for CK7 expression (Figure 1f).

**Figure 2:**
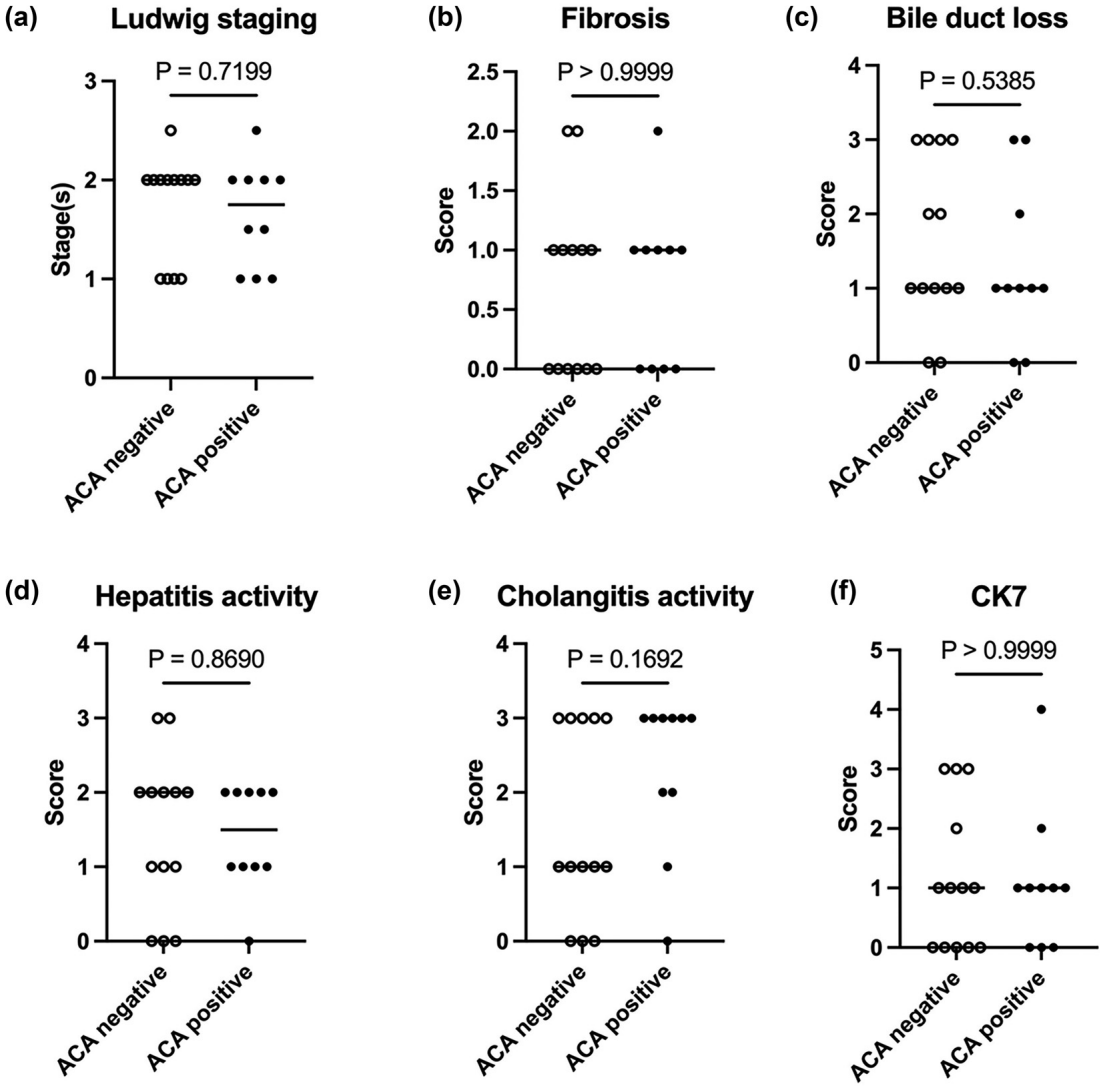
Comparison of Ludwig stages and Nakanuma scales of ALBI grade 1. (a) Ludwig stage. (b–e) Nakanuma scales. (f) CK7 score. Mann–Whitney *U* test was used.

**Figure 3:**
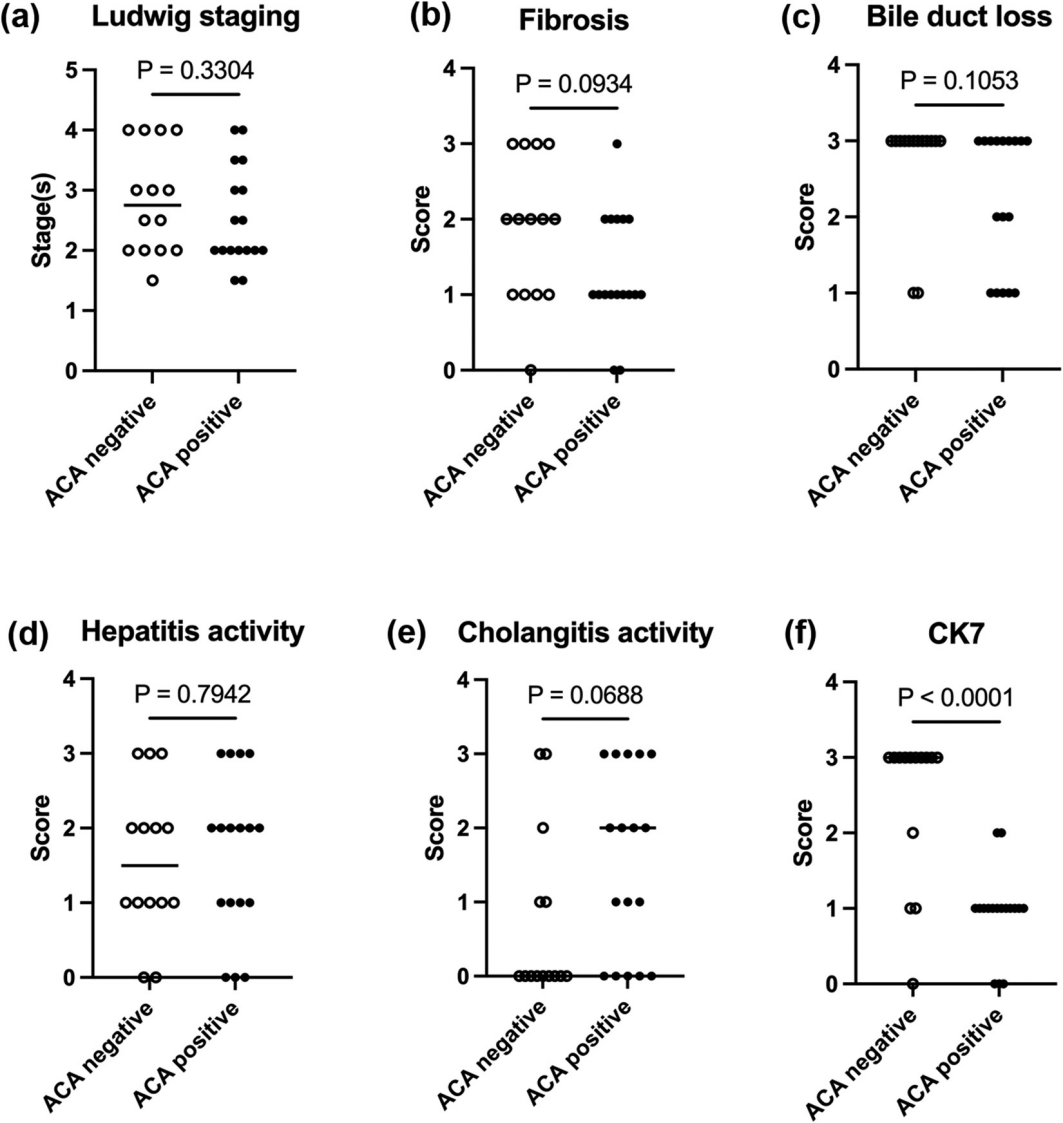
Comparison of Ludwig stages and Nakanuma scales of ALBI grade 2&3. (a) Ludwig stage. (b–e) Nakanuma scales. (f) CK7 score. Mann–Whitney *U* test was used.

To investigate the pathological differences in ALBI grading, we matched patients based on ALBI grade 1 and grades 2 and 3. In ALBI grade 1, the results were similar to the overall histological comparison between the two groups (Figure 2). When we focused on ALBI 2&3, CK7 expression showed obvious statistical significance between the two groups (Figure 3f).

## 4 Discussion

Although many previous studies have focused on the impact of autoimmune antibodies on PBC, research on the role of ACA is still limited. This research collected clinical indices from ACA-positive and ACA-negative PBC patients, investigating the differences between the two groups. Furthermore, we examined the gastroscopy results and liver cirrhosis status based on Child-Pugh grading and found significant differences in both observed variables between the groups. Finally, we semi-quantitatively analyzed liver biopsies to verify the histological differences between the two groups.

To begin with, like other autoimmune diseases, PBC onset shows a clear sex bias, which is more prevalent in females [21]. On this basis, our study further found that there was also a female-dominated gender tendency in the distribution of ACA in PBC patients (93.7% vs 86.3%). A report on ACA distribution in rheumatoid arthritis patients revealed a similar sex distribution bias [22]. There are plenty of explanations for this sex disequilibrium, and one of the major theories as to why autoimmune diseases are more prevalent in women is that they are related to autoantibodies. In fact, many studies have demonstrated that antibody levels are higher in women than in men. It has been suggested that the evolutionary perspective may be due to the fact that elevated antibodies in women can clear infections better in terms of reproduction to protect the mother and child, and thus increase the risk of autoimmune diseases [23]. Interestingly, other studies found that HLA genes play two sides of the same coin in immune system clearance of infections and autoimmune diseases [24]. Furthermore, Dumont-Lagacé et al. proved that sexual hormones affected the thymic epithelial cells, which may take part in the susceptibility to autoimmune diseases in females [25]. Markle et al. showed that sexual hormones could interfere with autoantibody generation and autoimmune disorder progression as well [26]. Yet, the direct mechanism of sex bias on PBC pathogenesis or ACA generation has not been discovered, studies in other autoimmune disorders shed light on follow-up studies in this area.

It has been reported controversial results on the prognostic value of ACA in several studies [27]. In our study, we chose to use cross-sectional bilirubin to assess prognosis. As one of the important prognostic indicators, the higher the bilirubin, the worse the prognosis of PBC. Studies have proven that liver transplantation and death risk increase as the disease progresses and bilirubin increases in patients with normal bilirubin at baseline [18,28]. Besides, ALBI grade was also used to evaluate the prognosis, which was first raised in the prognosis of hepatocellular carcinoma and further validated in PBC patients [13,14]. Overall, Bilirubin was remarkably lower in the ACA-positive group. Notably, further analysis demonstrated that this difference between bilirubin and ALBI was more significant in the cirrhotic group, and both were better in the ACA-positive group. These results suggest that patients in the ACA-positive group may have a relatively good prognosis.

In addition to bilirubin, there was another intriguing discovery in our study was that AST, ALT, and IgG were generally lower in the ACA-positive group than in the negative group. Although AST and ALT have less diagnostic and prognostic value than ALP, GGT, and bilirubin in PBC, their persistent elevation still reflects the degree of parenchymal inflammation in the liver. These parameters also involved several different UDCA response evaluation criteria, including Paris I and II and UK-PBC scores [1,29]. In this study, most of the patients were treated patients, and as can be seen from Table 1, the median level of AST was higher in the ACA negative group than in the positive group and was higher than 1.5*ULN, which may indicate a poorer UDCA response in ACA-negative patients. Besides, the AST/ALT ratio was comparable in the two groups (Table 1). According to the guideline, an AST/ALT ratio >1 signifies the progression of liver fibrosis [1]. This may suggest that both were at comparable levels in the progression of liver fibrosis for patients at this cross-section.

Though the hepatic venous pressure gradient is the most accurate method of diagnosing portal hypertension, not all patients undergo this assessment, particularly outpatients and those who are in the early stage of PBC, due to its invasive nature. A study conducted by Hu et al. showed that low PLT is the risk factor for pre-cirrhotic PBC, which we have also proven in our multi-variate analysis in non-cirrhosis patients [30]. Moreover, our study confirmed another risk factor – the existence of a positive correlation between ACA positivity and gastroesophageal varices in patients with non-cirrhotic PBC, which was consistent with previous finding that ACA positivity is a risk factor for portal hypertension in PBC patients by Nakamura et al. [27]. In patients with cirrhosis, the structural and vascular changes in the liver are more pronounced, and intrahepatic resistance is greatly increased, making them more susceptible to portal hypertension due to a variety of factors, which may obscure the association between ACA and portal hypertension, and therefore, we focused more on the non-cirrhotic phase of the disease.

**Figure 4:**
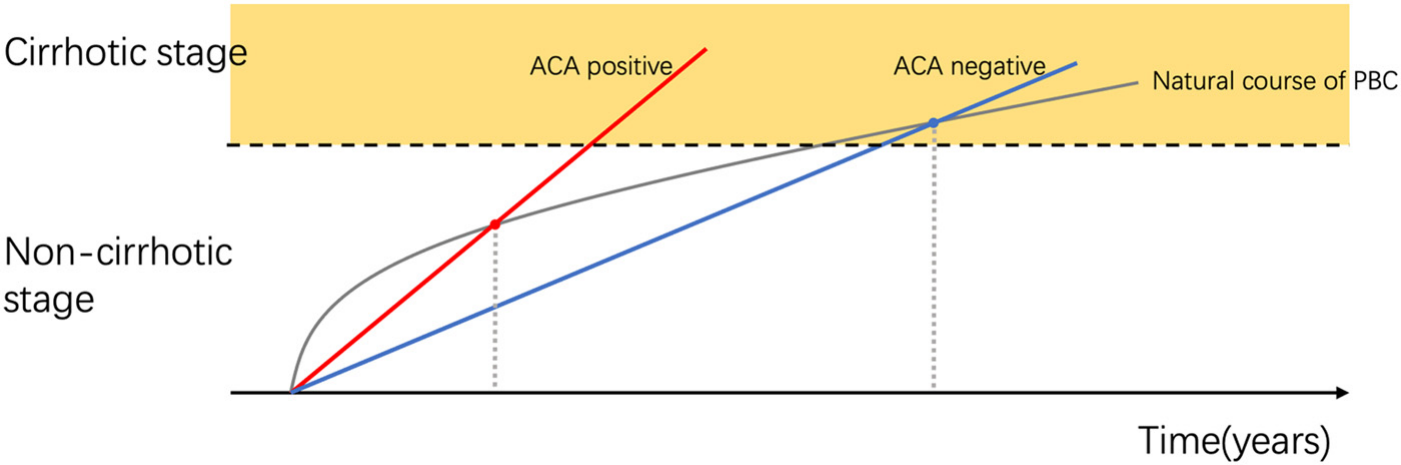
Assumed gastroesophageal varices progression pattern of PBC patients with positive and negative ACA. Gray curve: assumed natural course of PBC. Straight red line: assumed varix progression in ACA-positive PBC patients. Blue line: assumed varix progression of ACA-negative PBC patients. The dot where the red line and the curve intersect occurs earlier than the point where the blue line and the curve intersect.

Our results showed that there were little differences between the two groups in ALP and GGT, which are important indexes used in the diagnosis of PBC. Both ALP and GGT reflect the degree of cholestasis in the natural course of PBC in laboratory tests [3]. The weighted results of the CK7 histological results also did not reach statistical significance. In histology, CK7 positive could be regarded as the symbol of ductular reaction, which is a hallmark of severe PBC [31]. Based on this finding, we conjectured that the development of portal hypertension and gastroesophageal varices in ACA-positive PBC patients might not occur by interfering with the specific disease progress in ductular destruction of PBC cholestasis directly but may be facilitated by other autoimmune mechanisms. This may make the time point of development of portal hypertension in ACA-positive patients appear earlier during the PBC disease process (Figure 4). Moreover, a previous study by Nakamura et al. demonstrated that ACA-positivity is a risk factor for portal hypertension in PBC patients [27]. This has been observed similarly in other autoimmune diseases as well. In patients with systemic sclerosis, the presence of ACA is one of the risk factors for pulmonary hypertension [32]. Interestingly, ACA positivity can reach more than 70% in patients with systemic sclerosis and PBC overlap [33,34]. This also suggests that there may be a commonality in the course of ACA-associated elevated vascular pressure in these autoimmune diseases. There are few studies on the mechanism of ACA and portal hypertension and gastroesophageal varices in patients with PBC. Our study may provide some new ideas for future mechanism-related studies.

In addition, PLT showed a negative correlation with varix levels. Consequently, we examined the known comorbidities of portal hypertension in these patients. Liver diseases can cause increased vascular pressure, leading to spleen size increases (even splenomegaly), which triggers PLT phagocytosis and PLT count decreases. Accordingly, the PLT and WBC of the ACA-positive group were lower than those of the ACA-negative group.

In our observation of patients who were also in ALBI grades 2 and 3 on pathologic examination, the ACA-positive patients had considerably lower CK7 than the negative group, and when their ALP levels were compared, they were also substantially lower in the positive group (Table S1). Therefore, we hypothesized that a better level of prognosis may exist in ACA-positive patients with good portal hypertension monitoring. Combining our deduction above, this also implies that more optimal control of the disease can be achieved by earlier intervention in the management of portal hypertension in ACA-positive patients, albeit at an earlier stage of progression.

There are several limitations of this research. First, this was a cross-sectional study and does not provide a good representation of the changes in the dynamic progression of PBC. Second, this was a retrospective study, and thus, there are inherent biases in the data collection. Moreover, this study was only able to infer the reasons for the differences between the ACA-positive and ACA-negative groups based on the objective course of the disease and the observed pathological manifestations, and the underlying mechanism has not yet been elucidated. Nonetheless, this study still revealed the differences between ACA-positive and ACA-negative PBC patients in various aspects based on a large cohort of data, which were supported by pathological findings. Our study revealed that ACA is correlated with gastroesophageal varices and portal hypertension in PBC patients, which suggests patients’ specific management besides UDCA treatment.

## 5 Conclusion

In conclusion, we collected and compared biochemical information, gastroscopic findings, and liver biopsy analyses from ACA-positive and ACA-negative PBC patients. Combining multiple results, we present new insight into gastroesophageal varices in the natural PBC disease progression in ACA-positive patients during PBC. This will be a guide to capture disease progression in the future when diagnosing and treating this group of patients, and more studies are needed to validate this potential association.

## Supplementary Material

Supplementary Table
