# Supplementary material for: Gastroesophageal varices in primary biliary cholangitis with anti-centromere antibody positivity: Early onset?
Source: Open Life Sci. 2024 Nov 19;19(1):20220979. doi: 10.1515/biol-2022-0979 (PMC11588006; doi:10.1515/biol-2022-0979)
Supplement: Supplementary Table [file biol-2022-0979-sm.pdf]

Supplementary material

Table S1: Comparison of laboratory test parameters between 2 groups in liver biopsy patients

	ACA negative	ACA positive	P value
ALT (U/L)	61.8(98.25)	23.2(23.2)	<0.001
AST (U/L)	81.0(75.07)	37.0(30.25)	<0.001
TBIL (μmol/L)	33.7(34.03)	14.2(16.65)	0.028
GGT (U/L)	285.1(439.4)	168.5(296.45)	0.071
ALP (U/L)	290.5(296.7)	187.1(135.4)	0.015
ALB (g/L)	39.15(8.67)	37.4(9.45)	0.356
IgA (g/L)	3.46(2.01)	3.07(1.8)	0.863
IgG (g/L)	18.0(5.92)	14.4(5.2)	0.003
IgM (g/L)	4.09(1.20)	2.08(2.66)	0.013
WBC (*109/L)	4.93(2.39)	5.08(4.49)	0.276
PLT (*109/L)	175.5(129.25)	209(156.5)	0.747
PT (s)	11.0(1.35)	10.8(2.65)	0.958

Data were expressed in *n*(%) and median (interquartile). Abbreviations: alanine aminotransferase; AST: aspartate aminotransferase; TBIL: total bilirubin; GGT: gamma-glutamyltranspeptidase: ALP:alkaline phosphatase; ALB: albumin; WBC: white blood cell; PLT: platelet; PT: prothrombin time. S: seconds. Mann–Whitney U test were used for all parameters. Reference range: ALT 7–40 U/L; AST 13–35 U/L; TBIL 5–21 μmol/L; ALB 40–55 g/L; GGT 7–45 U/L; ALP 50–79 U/L; IgG 7.0–16.0 g/L; IgA 0.7–4.0 g/L; IgM 0.4–2.3 g/L; WBC 3.5–9.5 × 10^9/L; PLT 120–350 × 10^9/L; PT 9.9–12.8 s.
